# Definition, Burden, and Predictors of HIV-Associated Wasting and Low Weight in the OPERA Cohort

**DOI:** 10.1089/aid.2023.0048

**Published:** 2023-12-04

**Authors:** Michael B. Wohlfeiler, Rachel Palmieri Weber, Laurence Brunet, Javeed Siddiqui, Michael Harbour, Amy L. Phillips, Brooke Hayward, Jennifer S. Fusco, Ricky K. Hsu, Gregory P. Fusco

**Affiliations:** ^1^ AIDS Healthcare Foundation, Miami, Florida, USA.; ^2^ Epividian, Inc., Raleigh, North Carolina, USA.; ^3^ TeleMed2U, Roseville, California, USA.; ^4^ EMD Serono, Rockland, Massachusetts, USA.; ^5^ AIDS Healthcare Foundation, New York, New York, USA.; ^6^ NYU Langone Medical Center, New York, New York, USA.

**Keywords:** comorbidity, HIV wasting syndrome, incidence, prevalence, weight loss

## Abstract

We aimed to describe the prevalence, incidence, and predictors of HIV-associated wasting (HIVAW)/low weight among people with HIV (PWH) in the United States. We conducted an observational, clinical cohort analysis, utilizing prospectively collected electronic health record data obtained from the Observational Pharmaco-Epidemiology Research & Analysis (OPERA^®^) cohort. HIVAW/low weight included a wasting or low body–mass index (BMI)/underweight diagnosis (ICD codes and title search) or BMI <20 kg/m^2^. Prevalence was estimated among adult PWH in care from 2012 to 2015 and 2016 to 2020. Incidence from January 1, 2016, to October 31, 2021, was estimated using univariate Poisson regression among eligible PWH without prior HIVAW/low weight. Demographic and clinical predictors of incident HIVAW/low weight were included in multivariable logistic regression models, stratified by antiretroviral therapy (ART) experience. The period prevalence of HIVAW/low weight was 12% in both 2012–2015 and 2016–2020. Among 67,119 PWH without any prior HIVAW/low weight, 7% experienced incident HIVAW/low weight a median 64 months from HIV diagnosis. In multivariable regression models, similar predictor patterns were observed among ART-naïve and ART-experienced PWH without any prior HIVAW/low weight: lower odds of HIVAW/low weight with older age, female sex, Black race, and Hispanic ethnicity and higher odds with Medicaid. Notably, there was a dose–response relationship between increasing Veterans Aging Cohort Study Mortality Index scores and incident HIVAW/low weight in both groups. Wasting/low weight remains a challenge for PWH and may be underappreciated by providers. Advanced HIV and comorbidities significantly predict incident HIVAW/low weight. Increasing awareness of HIVAW, especially among frailer PWH, could improve the care of affected PWH.

## Introduction

HIV-associated wasting (HIVAW) was a frequently occurring syndrome at the beginning of the AIDS epidemic before the use of highly effective antiretroviral therapy (ART). In 1987, the Centers for Disease Control (CDC) included HIVAW as an end-of-life AIDS-defining illness and defined it as profound, involuntary weight loss of >10% of baseline body weight plus diarrhea or weakness with documented fever for ≥30 days in the absence of a concurrent illness.^1^ Subsequently, weight gain was attributed to an improvement in health, especially among severely immunocompromised people with HIV (PWH).^2–5^

While there is now growing concern that weight gain may be an undesired effect of ART,^6–23^ weight loss remains a concern. Indeed, among both ART-experienced virologically suppressed,^24^ and ART-naïve^25^ PWH with a normal baseline body–mass index (BMI) in the Observational Pharmaco-Epidemiology Research & Analysis (OPERA) cohort, 1%–3% transitioned to an underweight BMI after initiating ART or switching regimens. The relationship among HIV infection, ART, and changes in weight remains unresolved.

There is limited observational research on the prevalence and incidence of HIVAW over time. The Nutrition for Healthy Living (NFHL) study in the United States defined wasting as a loss of >10% baseline body weight, loss of >5% baseline body weight during a 6-month period with the loss sustained for ≥1 year, or BMI decreasing to <20 kg/m^2^. Among 466 PWH in the study, 14% reported wasting at study enrollment (1995–1999); the incidence proportion of wasting over follow-up (through 2005) was ∼34%.^26^

Among 2,502 PWH in the Multicenter AIDS Cohort Study, incidence rates of wasting syndrome (as defined by the CDC^1^) increased yearly up to 1995, after which they declined. However, the percentage of AIDS diagnoses attributed to wasting syndrome increased over time (from 5% in 1988–1990 to 19% in 1996–1999), likely because of a reduction in AIDS cases due to effective therapy.^27^ Between 2005 and 2007, HIVAW prevalence was estimated at 8% in a claims-based observational study of a managed care population in the United States.^28^

A more recent claims-based study of HIVAW between 2012 and 2018 in the United States reported a prevalence of 18%, most strongly associated with Medicaid coverage or hospitalization. The prevalence of HIVAW did not substantially vary between PWH with (18%) or without (19%) a claim for ART in the 12 months following their HIV diagnosis date.^29^ In both claims-based studies,^28,29^ HIVAW was defined using medical and pharmacy claims for weight loss and cachexia diagnoses, wasting treatments (e.g., appetite stimulants), or enteral/parenteral nutrition.

We sought to comprehensively evaluate HIVAW in a large, U.S.-based clinical cohort of PWH by first estimating the prevalence of HIVAW/low weight, overall and by payer type and calendar year, in both the historical (2012–2015) and modern ART (2016–2020) eras. In the modern ART era, we also aimed to estimate the incidence of HIVAW/low weight over follow-up and assess predictors of incident HIVAW/low weight among PWH without any prior HIVAW/low weight.

## Materials and Methods

### Study design and populations

An observational, clinical cohort analysis utilizing prospectively collected electronic health record (EHR) data obtained from PWH in the OPERA cohort was used to address the study objectives. At the time of this study (November 2021), OPERA included the EHR data of 140,817 PWH from over 100 clinic locations across 22 U.S. states and territories, representing ∼13% of people living with diagnosed infection in the United States.^30^

OPERA complies with all Health Insurance Portability and Accountability Act (HIPAA) and Health Information Technology for Economic and Clinical Health (HITECH) Act requirements, which expand upon the ethical principles detailed in the 1964 Declaration of Helsinki, and has received annual institutional review board (IRB) approval from Advarra IRB, including a waiver of informed consent and authorization for use of protected health information.

The prevalence of HIVAW/low weight was estimated during both the historical era (2012–2015) and the modern ART era (2016–2020). The study populations for the prevalence analyses included PWH, ≥18 years of age who were active in care (≥1 OPERA visit during the specified era). The study objectives related to incidence and predictors of HIVAW/low weight were also assessed during the modern ART era (2016–2020, with follow-up through October 31, 2021). The adult active in care PWH in the modern ART era additionally had no malignancy (except basal cell carcinoma [BCC] or squamous cell carcinoma [SCC] or *in situ* cancer) within 3 years before the date of eligibility (baseline), no AIDS-defining opportunistic infections (OIs)^31^ within 12 months before baseline, and no HIVAW/low weight before baseline for the incidence analyses.

### HIVAW/low weight definition

HIVAW/low weight was defined as a diagnosis of wasting, diagnosis of low BMI or underweight, or BMI measurement <20 kg/m^2^; diagnoses were identified through ICD codes and title searches. Instances where these criteria were met but were preceded by a malignancy within 3 years or an AIDS-defining OI within 12 months before the date of HIVAW/low weight were not classified as HIVAW/low weight to ensure that any HIVAW/low weight was not attributable to conditions that frequently result in wasting or significant weight loss.

### Statistical analyses

Prevalence was defined as the proportion of the total eligible study population that ever met the criteria for HIVAW/low weight during the specified era (i.e., historical or modern ART era). Univariate Poisson regression was employed to estimate the incidence rate (IR) and 95% confidence interval (CI) of HIVAW/low weight in the modern ART era. Person-time was censored at the first of the following events: incident HIVAW/low weight (outcome of interest), incident malignancy (except BCC, SCC, or *in situ* cancer), incident AIDS-defining OI, loss to follow-up (no clinical contact for >12 months), death, or study end (October 31, 2021).

The univariate distributions of baseline demographic and clinical characteristics were described among PWH who did and did not experience incident HIVAW/low weight. Predictors of incident HIVAW/low weight were evaluated with multivariable logistic regression models stratified by ART experience at baseline; demographic and clinical characteristics at baseline were selected for inclusion based on scientific literature and expert opinion. The predictor analysis was restricted to the subset of the population with complete covariate data (i.e., complete case analysis).

Since HIVAW is generally defined as progressive involuntary weight loss of both fat and lean tissue, we redefined incident HIVAW/low weight to include a criterion that might better reflect progressive weight loss for use in a sensitivity analysis. Analyses estimating incidence and evaluating predictors of HIVAW/low weight were repeated with a modified definition of incident HIVAW/low weight for the outcome among PWH without prior HIVAW/low weight according to the main study definition (i.e., the study populations remained the same between the main and sensitivity analyses). The redefined outcome included diagnoses of wasting or low BMI/underweight (as in the main study) as well as two consecutive BMI measurements <18.5 kg/m^2^ or loss of ≥10% of baseline body weight within 12 months.

## Results

### Prevalence of HIVAW/low weight

Of 140,817 PWH in OPERA, 59,026 were eligible during the historical era (2012–2015) and 101,026 were eligible during the modern ART era (2016–2020). The period prevalence of HIVAW/low weight was 12% in both eras; BMI measurements <20 kg/m^2^ accounted for most cases (Table 1).

**Table 1.** Prevalence Proportion (95% Confidence Interval) of People with HIV with HIV-Associated Wasting/Low Weight in the Historical (2012–2015) and Modern Antiretroviral Therapy (2016–2020) Eras

	Historical era (2012–2015) *N* = 59,026	Modern ART era (2016–2020) *N* = 101,026
Overall	12.2 (11.9, 12.4)	11.9 (11.7, 12.1)
Payer		
Medicaid	15.3 (14.7, 15.9)	14.9 (14.4, 15.4)
Medicare	14.7 (13.9, 15.5)	14.9 (14.2, 15.6)
Commercial insurance	10.4 (10.0, 10.9)	10.7 (10.4, 11.0)
ADAP/Ryan White	11.8 (11.3, 12.3)	11.6 (11.2, 12.0)
Other	11.5 (10.3, 12.7)	10.7 (10.0, 11.5)
No payer info	11.5 (10.9, 12.1)	11.2 (10.7, 11.7)

ADAP, AIDS Drug Assistance Program; ART, antiretroviral therapy.

Prevalence did not substantially vary by calendar year; the per calendar year prevalence ranged from 7.2% (95% CI: 6.9%–7.5%) in 2012 to 8.3% (95% CI: 8.0%–8.5%) in 2015 during the historical era and 5.2% (95% CI: 5.0%–5.4%) in 2020 to 7.9% (95% CI: 7.7%–8.2%) in 2016 during the modern ART era. The prevalence of HIVAW/low weight was higher among PWH who reported having Medicare or Medicaid as payer compared with PWH who reported other payers (e.g., commercial insurance) (Table 1).

### Incidence of HIVAW/low weight

Among 67,119 PWH without prior HIVAW/low weight in the modern ART era, 4,962 (7%) experienced incident HIVAW/low weight a median 9 months (interquartile range [IQR]: 1, 24) from baseline (IR: 2.20 per 100 person-years [py], 95% CI: 2.14–2.27) and a median 64 months (IQR: 14, 174) from the date of HIV diagnosis (IR: 0.66 per 100 py, 95% CI: 0.64–0.68).

Compared with PWH who did not experience incident HIVAW/low weight, PWH with incident HIVAW/low weight were more likely to be Black, to be receiving HIV care in the Southern region of the United States, or to report having Medicaid or Medicare as payer, and less likely to be Hispanic or to report having commercial insurance or the AIDS Drug Assistance Program/Ryan White Program as payer (Table 2).

**Table 2.** Baseline Demographic and Clinical Characteristics of People with HIV in the Modern Antiretroviral Therapy Era (January 2016–October 2021), by Incident HIV-Associated Wasting/Low Weight Experience Over Follow-Up

Baseline^a^ demographic or clinical characteristic	PWH with incident HIVAW/low weight *N* = 4,962	PWH without incident HIVAW/low weight *N* = 62,157
Median age, years (IQR)	40 (28, 53)	41 (31, 52)
Female sex, *n* (%)	926 (19)	11,389 (18)
Black race, *n* (%)	2,559 (52)	28,655 (46)
Hispanic ethnicity, *n* (%)	805 (16)	13,708 (22)
Southern U.S. geographic region, *n* (%)	3,555 (72)	41,781 (67)
Payer^b^, *n* (%)		
Medicaid	1,234 (25)	13,858 (22)
Medicare	535 (11)	5,412 (9)
Commercial insurance	1,828 (37)	27,023 (43)
ADAP/Ryan White Program	1,274 (26)	17,949 (29)
Median VACS Mortality Index^32^ score (IQR)	20 (10, 34)	13 (6, 24)
Median months between HIV diagnosis and baseline (IQR)	47 (1, 157)	57 (2, 156)
Median HIV viral load (copies/mL) (IQR)	70 (19, 36,170)	20 (19, 7,420)
Median eGFR^c^ (mL/min/1.73 m^2^) (IQR)	103 (84, 120)	99 (83, 115)
Comorbidities, *n* (%)	2,333 (47)	32,918 (53)

^a^ First date in 2016–2020 where the individual was HIV+, 18 years of age or older, and had an active OPERA^®^ visit.

^b^ Payer data are not mutually exclusive; PWH can have more than one payer at a time.

^c^ Calculated using the Chronic Kidney Disease Epidemiology Collaboration (CKD-EPI) equation (2009).

ADAP, AIDS Drug Assistance Program; eGFR, estimated glomerular filtration rate; HIVAW, HIV-associated wasting; IQR, interquartile range; PWH, people with HIV; VACS, Veterans Aging Cohort Study.

PWH who experienced incident HIVAW/low weight had a higher median Veterans Aging Cohort Study (VACS) Mortality Index score^32^ at baseline and were less likely to have any comorbidities than PWH who did not experience incident HIVAW/low weight (Table 2).

### Predictors of HIVAW/low weight

Of 11,525 ART-naïve PWH and 39,166 ART-experienced PWH without prior HIVAW/low weight and with complete predictor data, 1,152 (10%) and 2,306 (6%), respectively, experienced incident HIVAW/low weight over follow-up. Similar predictor patterns were observed from the multivariable regression models among both groups. There were lower odds of incident HIVAW/low weight with older age, female sex, Black race, and Hispanic ethnicity, and higher odds with Medicaid coverage.

There was a dose–response relationship with increasing VACS Mortality Index scores associated with higher odds of HIVAW/low weight in both groups. The adjusted odds ratios (aORs) of incident HIVAW/low weight were 1.29 (95% CI: 1.09–1.52), 1.81 (95% CI: 1.46–2.25), and 2.94 (95% CI: 2.28–3.80) for ART-naïve PWH with baseline VACS scores of 15 to <30, 30 to <45, and ≥45, respectively, when compared with ART-naïve PWH with a VACS score <15. Among ART-experienced PWH, the results were similar (aORs of 1.36, 1.88, and 2.41, respectively).

**FIG. 1.**
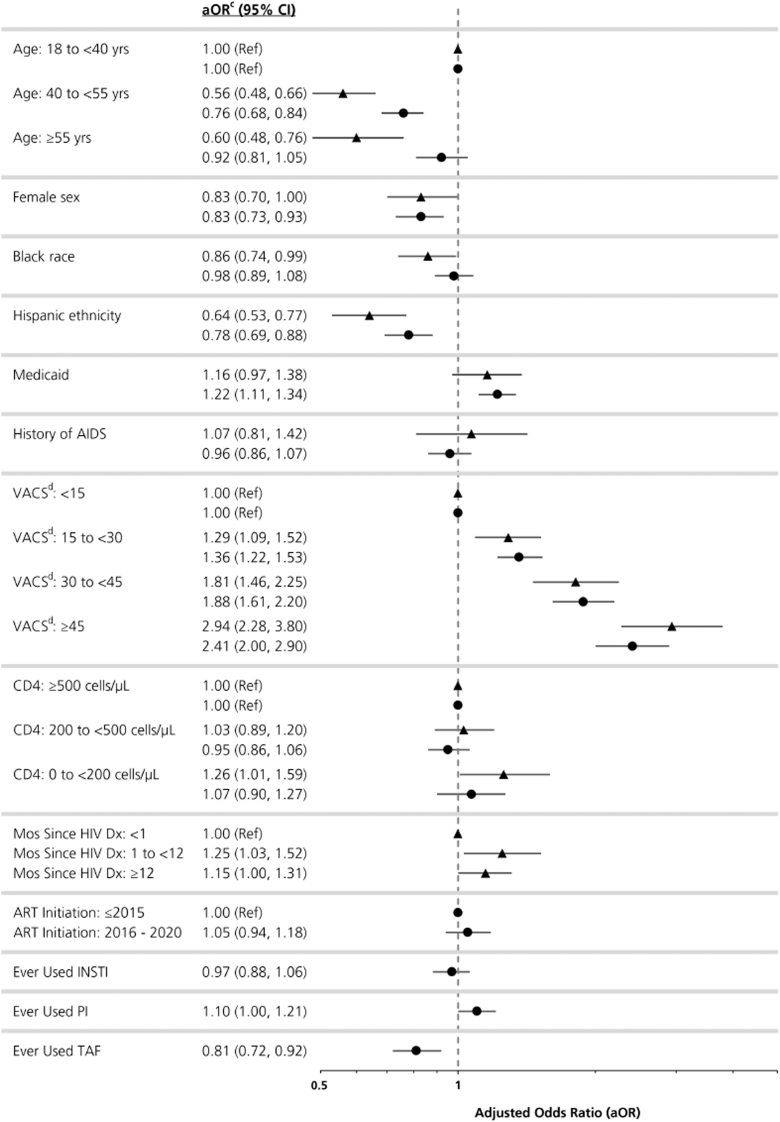
Predictors of incident HIV-associated wasting/low weight among ART-naïve (*triangle*; *N* = 11,525)^a^ and ART-experienced (*circle*; *N* = 39,166)^b^ people with HIV in OPERA^®^ (January 2016–October 2021). ^a^A total of 3,507 (23%) ART-naïve PWH were excluded from the model due to missing data in the following variables: sex (*n* = 5; < 1%), race (*n* = 426; 3%), ethnicity (*n* = 479; 3%), CD4 cell count (*n* = 1,941; 13%), and VACS score (*n* = 3,315; 22%). ^b^A total of 7,865 (17%) ART-experienced PWH were excluded from the model due to missing data in the following variables: sex (*n* = 4; < 1%), race (*n* = 1,425; 3%), ethnicity (*n* = 1,529; 3%), CD4 cell count (*n* = 3,219; 7%), and VACS score (*n* = 7,251; 15%). ^c^Adjusted for all variables in the model. ^d^VACS Mortality Index: composite index used to estimate a 5-year risk of all-cause mortality; a higher VACS score is associated with a higher risk of mortality.^32^ aOR, adjusted odds ratio; AIDS, acquired immunodeficiency syndrome; ART, antiretroviral therapy; CI, confidence interval; Dx, diagnosis; INSTI, integrase strand transfer inhibitor; PI, protease inhibitor; PWH, people living with HIV; VACS, Veterans Aging Cohort Study.

Finally, tenofovir alafenamide (TAF) was associated with an almost 20% decrease in the odds of incident HIVAW/low weight (aOR: 0.81, 95% CI: 0.72–0.92) among ART-experienced PWH (Fig. 1 and Supplementary Tables S1 and S2).

### Sensitivity analysis

We redefined incident HIVAW/low weight to include a criterion that might better reflect progressive weight loss. In addition to a wasting or low BMI/underweight diagnosis (text or code), redefined incident HIVAW/low weight also included two consecutive BMI measurements <18.5 kg/m^2^ or loss of ≥10% of baseline body weight within 12 months. A majority of PWH met only one of the criteria, most commonly a loss of ≥10% of body weight within 12 months of baseline; a total of 4,542 (7%) PWH experienced redefined HIVAW/low weight a median 7 months (IQR: 3, 11) and 79 months (IQR: 16, 187) from baseline and HIV diagnosis, respectively.

Results were mostly consistent with the main study analysis that evaluated predictors of incident HIVAW/low weight among ART-naïve (Supplementary Table S1) and ART-experienced PWH (Supplementary Table S2). There remained a dose–response relationship with increasing VACS scores associated with higher odds of redefined HIVAW/low weight, although results were slightly attenuated from the main analysis.

Female sex was associated with a decrease in odds of incident HIVAW/low weight in the main study analysis, but an increase in odds in the sensitivity analysis, for both ART-naïve (main: aOR 0.83, 95% CI: 0.70–1.00; sensitivity: aOR 1.29, 95% CI: 1.07–1.56) and ART-experienced (main: aOR 0.83, 95% CI: 0.73–0.93; sensitivity: aOR 1.12, 95% CI: 1.01–1.24) PWH.

## Discussion

We comprehensively evaluated HIVAW/low weight (wasting or low BMI/underweight diagnosis or a BMI <20 kg/m^2^) in the U.S.-based, OPERA, observational clinical cohort. The prevalence of HIVAW/low weight was 12% in both the historical (2012–2015) and modern ART (2016–2020) eras and did not vary substantially by calendar year.

Among 67,119 PWH without prior HIVAW/low weight in the modern ART era, 7% experienced incident HIVAW/low weight over follow-up, a median 64 months from HIV diagnosis. Similar predictor patterns from multivariable logistic regression models were observed among both ART-naïve and ART-experienced PWH; most notably, there was a significant dose–response relationship with increasing VACS scores associated with higher odds of HIVAW/low weight.

In observational research, a range of HIVAW prevalence and incidence estimates have been reported. The variability is likely due to the variety of criteria used to identify HIVAW in the absence of objective measures or a standard definition. Additionally, the studies were conducted over multiple decades. Of only four studies that assessed the prevalence or incidence of HIVAW in the United States, two were conducted early in the HIV epidemic (before 2000).

In the Multicenter AIDS Cohort, the incidence of wasting syndrome (according to CDC criteria) increased from 7.5 (1988–1990) to 22.1 per 1,000 py (1994–1995), but decreased to 13.4 per 1,000 py in 1996–1999^27^; in the OPERA cohort, the incidence of HIVAW/low weight was higher (22 per 1,000 py) in January 2016–October 2021. As in this study, the NFHL study^26,33^ included a BMI <20 kg/m^2^ as part of their wasting criteria; at study start in 1995, the prevalence of self-reported wasting was 14%, similar to the 12% we reported in the OPERA cohort beginning in 2012.

However, more than a third of the NFHL cohort with sufficient follow-up had lost 5%–10% of their body weight or reported a BMI <20 kg/m^2^ compared with the 7% of PWH who experienced incident HIVAW/low weight in this study. Finally, in more recent studies, the prevalence estimates of HIVAW in two claims-based studies were 8% (January 2005–July 2007)^28^ and 18% (July 2012–September 2018),^29^ which straddle the 12% prevalence estimates in this study (2012–2015 and 2016–2020).

Only one other recent study has assessed predictors of HIVAW: the IBM MarketScan medical and pharmacy claims study ranging from July 2012 to September 2018.^29^ The HIVAW criteria and data sources were different from this study and included claims related to nutritional marasmus, abnormal or unintentional weight loss, cachexia, or BMI <19 kg/m^2^; claims for treatment (appetite stimulants and nontestosterone anabolic agents); evidence of enteral or parenteral nutrition; or a combination of claims for diagnosis and treatment of wasting.

With respect to age, the investigators noted a 1% increase in the odds of HIVAW per year of age. In this study, the association between age and HIVAW/low weight varied with the HIVAW/low weight definition used. The definition used for our main analysis observed a protective effect with age, which likely was the result of misclassifying thinner, young PWH as having HIVAW/low weight. However, consistent with the claims study, there was a numerical increase in the odds of redefined HIVAW/low weight with increasing age in our sensitivity analysis.

With respect to sex, the claims study reported an increased odds of HIVAW with male sex (OR: 1.24, 95% CI: 1.17–1.31); in this study, results for the association with sex were consistent with the claims study in the main analysis, but opposite in sensitivity analyses. The differences between and within studies (i.e., different definitions of HIVAW) offer insight into how important it is to consider multiple avenues through which HIVAW may be identified in observational research and in clinical settings, especially in the absence of a standard definition.

Of note, there was a significant association between HIVAW and Medicaid coverage in both the claims study (71% higher odds of HIVAW) and this study (main: aOR 1.22, 95% CI: 1.11–1.34; sensitivity: aOR 1.25, 95% CI: 1.15–1.37). PWH with Medicaid coverage are more likely to have lower income levels, which may result in food insecurity, and disability, which can sometimes reflect a higher disease burden^34^; these characteristics potentially contribute to HIVAW/low weight.

Additionally, the claims study reported a 9% increase in the odds of HIVAW with each point increase in the Charlson Comorbidity Index, which was developed to predict mortality by measuring disease burden (i.e., by weighting select comorbidities)^35,36^; PWH with HIVAW had higher proportions of cardiovascular and pulmonary diseases, diabetes, and renal disease than PWH without HIVAW.

Increasing VACS scores,^32^ which take into account typical predictors of mortality among PWH (age, CD4 cell count, and viral load) as well as markers of organ system injury (hepatic and renal), were significantly associated with an increase in the odds of HIVAW/low weight in this study. These robust findings of associations with Medicaid coverage and markers of comorbidity and HIV disease progression with HIVAW across data sources, populations, and definitions of HIVAW may highlight subsets of PWH who are potentially more vulnerable to developing HIVAW.

Potential misclassification of HIVAW/low weight is the primary limitation of any study of HIVAW. While HIVAW is generally defined as progressive, involuntary weight loss with both fat and lean muscle tissue loss, 75% of the PWH who experienced incident HIVAW/low weight in this study met the definition by experiencing a single BMI <20 kg/m^2^ or a low BMI/underweight diagnosis. Of those, only 6% received a subsequent wasting diagnosis.

While the wasting diagnosis inherently includes progressive weight loss, a single BMI <20 kg/m^2^ or low BMI/underweight diagnosis may not. In sensitivity analyses, we redefined HIVAW/low weight to incorporate potentially meaningful weight loss (≥10% of baseline body weight within 12 months) and to identify PWH who more consistently had an underweight BMI (two consecutive BMI measurements <18.5 kg/m^2^) as criteria for incident HIVAW/low weight.

Additionally, involuntary weight loss was not captured in either definition, except when implied by a wasting diagnosis. Because HIVAW is not limited to underweight bodies, the definition we used in the main study analyses could potentially overrepresent younger PWH, who are naturally thinner, and underrepresent females who tend to have higher BMI.

The definition we used in sensitivity analyses allowed for PWH of all body sizes to be identified as potentially experiencing HIVAW, highlighting unusual weight loss over time. Without an objective measure or standard criteria for identifying HIVAW, caution must be taken when using body size as a criterion for wasting, especially since wasting can occur at all sizes.

The role of ART and weight gain,^6–23^ a mechanism that is not well understood, must also be considered. In this study, TAF use was associated with an almost 20% decrease in the odds of incident HIVAW/low weight among ART-experienced PWH. However, someone who used TAF and experienced weight gain is less likely to meet our definitions of HIVAW/low weight, which relied heavily on low BMI.

Outside of the challenge of identifying HIVAW, this study may also be limited by missing data in the predictor analysis. There were 5,506 (7%) PWH who were identified as ART-experienced in their EHR, but who had no ART records in the OPERA database. An additional 3,507 (23%) ART-naïve PWH and 7,865 (17%) ART-experienced PWH were excluded from the predictor analysis, mostly due to missing CD4 cell counts or VACS scores at baseline. It is unclear whether PWH missing those data are more likely to be healthier or sicker than the PWH included in the complete case analysis, making it difficult to speculate the potential direction(s) of bias in the results.

This study, however, does have several strengths. It is one of a few observational studies evaluating HIVAW among PWH and only one of two studies focused on the most recent years of the modern ART era.

The OPERA cohort is large and diverse, representing ∼13% of PWH in the United States,^30^ and is the only recent study to use EHR data to evaluate HIVAW. The OPERA cohort yielded substantial study populations of 59,026 PWH in 2012–2015 and 101,026 PWH (67,199 of which had no prior HIVAW/low weight) in 2016–2020, thus conferring adequate power for statistical assessments of HIVAW and generalizability of the population.

The richness of the prospectively captured clinical data of this large population of PWH allowed for a comprehensive assessment of the burden and predictors of HIVAW/low weight; the breadth of the EHR data, especially in the availability of BMI measurements over time, allowed for sensitivity analyses related to the definition of HIVAW. Efforts were made to exclude weight loss associated with comorbidities such as OIs and invasive cancer in both eligibility for the study and when the outcome of HIVAW/low weight was identified.

Between January 2016 and October 2021, incident HIVAW/low weight was identified in 7% of a large population of PWH in the United States. Although not common, HIVAW remains a challenge for PWH and may be underappreciated by health care providers based on a large proportion of PWH without a diagnosis of wasting in this study. Definitions of HIVAW may be surrogates for true HIVAW in the absence of an objective measure such as a diagnostic test or laboratory result.

Despite the difficulty in defining HIVAW, the VACS Mortality Index was the strongest predictor of HIVAW/low weight in both ART-naïve and ART-experienced PWH in both the main and sensitivity analyses, highlighting the relationship between advanced HIV and comorbidities with wasting. Particular attention must be paid to the potential for wasting, especially among frailer PWH. Increasing awareness of HIVAW could improve the care and clinical outcomes of PWH in an era of weight gain associated with ART.

## Supplementary Material

Supplemental data

Supplemental data

## Data Availability

The datasets used in this study are not publicly available due to privacy concerns and the proprietary nature of the database, but can be accessed upon reasonable request through the corresponding author to the OPERA Epidemiology and Clinical Advisory Board. Access to codes may be granted upon request, with parties agreeing to privacy restrictions and technological specifications and requirements.
