# Supplementary material for: Definition, Burden, and Predictors of HIV-Associated Wasting and Low Weight in the OPERA Cohort
Source: AIDS Res Hum Retroviruses. 2023 Dec 4;39(12):636–43. doi: 10.1089/aid.2023.0048 (PMC10712360; doi:10.1089/aid.2023.0048)
Supplement: Supplemental data [file Supp_TableS1.pdf]

Supplemental Table 1. Predictors of incident HIVAW/low weight as defined by the main study and in sensitivity analysis among ART-naïve people with HIV with and without incident HIVAW/low weight in the modern ART era (January 2016-October 2021)

Predictor at baseline[a], n (%)	MAIN STUDY ANALYSIS				SENSITIVITY ANALYSIS		
	With incident HIVAW/low weight[b] N = 1,152	Without incident HIVAW/low weight[b] N = 10,373	aOR[d] (95% CI)		With redefined incident HIVAW/low weight[c] N = 781	Without redefined incident HIVAW/low weight[c] N = 10,794	aOR[d] (95% CI)
Age, years							
18 to < 40	811 (70)	6,627 (64)	Reference		478 (61)	6,998 (65)	Reference
40 to < 55	230 (20)	2,729 (26)	0.56 (0.48, 0.66)		212 (27)	2,756 (26)	0.95 (0.80, 1.14)
≥ 55	111 (10)	1,017 (10)	0.60 (0.48, 0.76)		91 (12)	1,040 (10)	0.90 (0.69, 1.16)
Female sex	186 (16)	1,700 (16)	0.83 (0.70, 1.00)		178 (23)	1,712 (16)	1.29 (1.07, 1.56)
Black race	670 (58)	5,669 (55)	0.86 (0.74, 0.99)		454 (58)	5,906 (55)	0.94 (0.79, 1.12)
Hispanic ethnicity	174 (15)	2,216 (21)	0.64 (0.53, 0.77)		127 (16)	2,267 (21)	0.77 (0.62, 0.97)
Medicaid	171 (15)	1,315 (13)	1.16 (0.97, 1.38)		133 (17)	1,358 (13)	1.26 (1.03, 1.54)
History of AIDS	65 (6)	408 (4)	1.07 (0.81, 1.42)		47 (6)	428 (4)	1.22 (0.88, 1.68)
VACS Mortality Index							
0 to < 15	431 (37)	5,120 (49)	Reference		300 (38)	5,275 (49)	Reference
15 to <30	307 (27)	3,006 (29)	1.29 (1.09, 1.52)		211 (27)	3,115 (29)	1.14 (0.93, 1.38)
30 to < 45	179 (16)	1,259 (12)	1.81 (1.46, 2.25)		106 (14)	1,341 (12)	1.30 (1.00, 1.70)
≥ 45	235 (20)	988 (10)	2.94 (2.28, 3.80)		164 (21)	1,063 (10)	2.54 (1.88, 3.43)
CD4 cell count, cells/μL							
≥ 500	449 (39)	5,100 (49)	Reference		332 (43)	5,230 (49)	Reference
200 to < 500	420 (36)	3,914 (38)	1.03 (0.89, 1.20)		284 (36)	4,076 (38)	1.01 (0.84, 1.21)
0 to < 200	283 (25)	1,359 (13)	1.26 (1.01, 1.59)		165 (21)	1,488 (14)	0.98 (0.74, 1.28)
Months between HIV diagnosis & baseline							
< 1	462 (40)	4,311 (41)	Reference		304 (39)	4,487 (42)	Reference
1 to < 12	159 (14)	1,242 (12)	1.25 (1.03, 1.52)		117 (15)	1,292 (12)	1.37 (1.09, 1.71)
≥ 12	531 (46)	4,820 (47)	1.15 (1.00, 1.31)		360 (46)	5,015 (46)	1.06 (0.90, 1.25)

AIDS, acquired immunodeficiency syndrome; aOR, adjusted odds ratio; ART, antiretroviral therapy; CI, confidence interval; HIV, human immunodeficiency virus; HIVAW, HIV-associated wasting; μl, microliter; n, number; PWH, people living with HIV; TAF, tenofovir alafenamide; VACS, Veterans Aging Cohort Study

[a] Baseline = First date in 2016-2020 where the individual was HIV+, 18 years of age or older, and had an active OPERA® visit

[b] Incident HIVAW/low weight: Wasting or low BMI/underweight diagnosis (ICD codes, title search) or a BMI measurement <20 kg/m^2

[c] Redefined incident HIVAW/low weight: Wasting or low BMI/underweight diagnosis (ICD codes, title search), two consecutive BMI measurements <18.5 kg/m^2, or loss of ≥10% of baseline body weight within 12 months of baseline

[d] Adjusted for all variables in the table except where otherwise note
