# Supplementary material for: Definition, Burden, and Predictors of HIV-Associated Wasting and Low Weight in the OPERA Cohort
Source: AIDS Res Hum Retroviruses. 2023 Dec 4;39(12):636–43. doi: 10.1089/aid.2023.0048 (PMC10712360; doi:10.1089/aid.2023.0048)
Supplement: Supplemental data [file Supp_TableS2.pdf]

Supplemental Table 2. Predictors of incident HIVAW/low weight as defined by the main study and in sensitivity analysis among ART-experienced people with HIV with and without incident HIVAW/low weight in the modern ART era (January 2016-October 2021)

Predictor at baseline[a], n (%)	MAIN ANALYSIS			SENSITIVITY ANALYSIS		
	With incident HIVAW/low weight[b] N = 2,306	Without incident HIVAW/low weight[b] N = 36,860	aOR[d] (95% CI)	With redefined incident HIVAW/low weight[c] N = 2,547	Without redefined incident HIVAW/low weight[c] N = 36,639	aOR[d] (95% CI)
Age, years						
18 to < 40	947 (41)	15,414 (42)	Reference	912 (36)	15,461 (42)	Reference
40 to < 55	790 (34)	14,560 (40)	0.76 (0.68, 0.84)	1,018 (40)	14,336 (39)	1.04 (0.94, 1.14)
≥ 55	569 (25)	6,886 (19)	0.92 (0.81, 1.05)	617 (24)	6,842 (19)	1.12 (0.99, 1.28)
Female sex	443 (19)	6,823 (19)	0.83 (0.73, 0.93)	584 (23)	6,683 (18)	1.12 (1.01, 1.24)
Black race	1,154 (50)	16,926 (46)	0.98 (0.89, 1.08)	1,239 (49)	16,849 (46)	0.93 (0.85, 1.02)
Hispanic ethnicity	413 (18)	8,513 (23)	0.78 (0.69, 0.88)	468 (18)	8,459 (23)	0.79 (0.70, 0.89)
Medicaid	757 (33)	10,039 (27)	1.22 (1.11, 1.34)	868 (34)	9,930 (27)	1.25 (1.14, 1.36)
History of AIDS	579 (25)	8,341 (23)	0.96 (0.86, 1.07)	656 (26)	8,260 (23)	0.96 (0.87, 1.06)
VACS Mortality Index						
0 to < 15	960 (42)	19,713 (53)	Reference	1,125 (44)	19,563 (53)	Reference
15 to <30	705 (31)	10,890 (30)	1.36 (1.22, 1.53)	761 (30)	10,838 (30)	1.12 (1.01, 1.25)
30 to < 45	343 (15)	3,768 (10)	1.88 (1.61, 2.20)	333 (13)	3,779 (10)	1.37 (1.18, 1.59)
≥ 45	298 (13)	2,489 (7)	2.41 (2.00, 2.90)	328 (13)	2,459 (7)	2.04 (1.71, 2.43)
CD4 cell count, cells/μL						
≥ 500	1,316 (57)	23,104 (63)	Reference	1,526 (60)	22,908 (63)	Reference
200 to < 500	701 (30)	10,824 (29)	0.95 (0.86, 1.06)	733 (29)	10,798 (29)	0.93 (0.84, 1.03)
0 to < 200	289 (13)	2,932 (8)	1.07 (0.90, 1.27)	288 (11)	2,933 (8)	1.01 (0.86, 1.20)
Calendar year ART initiation						
≤ 2015	1,373 (60)	21,291 (58)	Reference	1,585 (62)	21,075 (58)	Reference
2016 – 2020	933 (40)	15,569 (42)	1.05 (0.94, 1.18)	962 (38)	15,564 (42)	1.02 (0.91, 1.13)
Ever used INSTI	1,398 (61)	22,859 (62)	0.97 (0.88, 1.06)	1,515 (59)	22,755 (62)	0.97 (0.89, 1.06)
Ever used PI	825 (36)	11,445 (31)	1.10 (1.00, 1.21)	954 (37)	11,318 (31)	1.13 (1.03, 1.24)
Ever used TAF[e]	549 (24)	10,118 (27)	0.81 (0.72, 0.92)	550 (22)	10,135 (28)	0.78 (0.69, 0.88)

AIDS, acquired immunodeficiency syndrome; aOR, adjusted odds ratio; ART, antiretroviral therapy; CI, confidence interval; HIV, human immunodeficiency virus; HIVAW, HIV-associated wasting; μl, microliter; n, number; PWH, people living with HIV; TAF, tenofovir alafenamide; VACS, Veterans Aging Cohort Study

[a] Baseline = First date in 2016-2020 where the individual was HIV+, 18 years of age or older, and had an active OPERA® visit

[b] Incident HIVAW/low weight: Wasting or low BMI/underweight diagnosis (ICD codes, title search) or a BMI measurement <20 kg/m^2

[c] Redefined incident HIVAW/low weight: Wasting or low BMI/underweight diagnosis (ICD codes, title search), two consecutive BMI measurements <18.5 kg/m^2, or loss of ≥10% of baseline body weight within 12 months of baseline

[d] Adjusted for all variables in the table except where otherwise noted

[e] Including as part of pre-exposure prophylaxis (PrEP)
